# Changes in Social Behavior Over Time During the COVID-19 Pandemic

**DOI:** 10.7759/cureus.10754

**Published:** 2020-10-01

**Authors:** Megan M Sheehan, Elizabeth Pfoh, Sidra L Speaker, Michael Rothberg

**Affiliations:** 1 Center for Value-Based Care Research, Cleveland Clinic, Cleveland, USA

**Keywords:** covid-19, social distancing, public health

## Abstract

Public health recommendations aimed at limiting the spread of SARS-CoV-2 have encouraged social distancing and masks as economies across the United States re-open. Understanding adherence to these guidelines will inform further efforts to reduce transmission. In this repeated cross-sectional survey study, we describe changes in social behavior in Ohio during periods of declining and rising cases. While essential activities remained consistent over time, more individuals attended gatherings of 10 or more people as cases rose, particularly in the 18-29 age group. A majority of individuals wore masks. It appears necessary to continue limiting gatherings and encourage mask-wearing, particularly among younger groups.

## Introduction

In March 2020, Ohio and other states closed schools, workplaces, and gathering spots to limit the transmission of SARS-CoV-2. Experience from prior pandemics and containment strategy modeling contributed to the development and implementation of these interventions [1-2]. Early studies from the current pandemic support their efficacy - a population-based cohort study from China showed that social distancing measures, home isolation, and improved medical resources were associated with a reduction in cases [3]. In a study of four metropolitan areas throughout the United States, community mitigation policies, including social distancing and personal protective measures, also correlated with declining cases [4]. A study of patient adherence to public health recommendations in three countries found that government response efforts positively influenced adherence [5]. In Ohio, gatherings of more than 10 people were largely prohibited, and the adoption of social distancing and cloth face masks was encouraged to prevent the rapid spread of the virus [6]. As states flattened the curve of new transmissions, they began to reopen. Unfortunately, as restrictions lifted, cases have increased [7]. Understanding to what extent individuals adhered to public health recommendations during reopening can help inform current efforts to shape public behavior and reduce transmission. In this study, we describe social behavior in Ohio early in the re-opening phase, when cases were still declining, and then afterward as cases rose.

## Materials and methods

**Figure 1:**
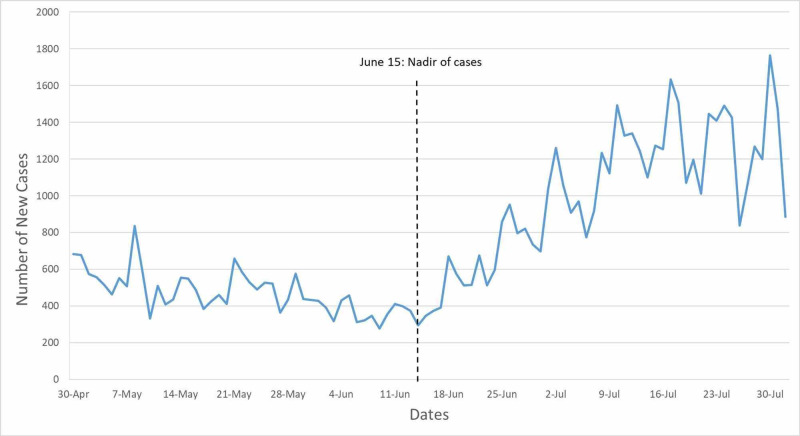
COVID-19 cases in Ohio

This repeated cross-sectional study surveyed adults who were seen at a large integrated health system in Ohio in the past 12 months and who are active on MyChart. MyChart is a personal health record (patient portal) that enables patients to access their health information online via their phone, tablet, or computer. We sent the survey via MyChart because it enabled us to identify a large population of patients and securely send a survey. We excluded patients who tested positive for SARS-COV2 before the survey. The survey was sent to 9,097 patients who had a visit to an internal medicine or family medicine physician in the prior year. Patients were sent a 14-question survey via MyChart about social distancing and hygiene behaviors in the past seven days [8]. The survey included questions about hand-washing, mask-wearing, essential activities, such as grocery shopping and seeking healthcare, and social activities such as attending public or private gatherings. The survey also asked about chronic health problems such as lung disease, immunosuppression, and high blood pressure. It was developed based on expert opinion and prior surveys [9-10]. It was pilot-tested with 10 individuals. After testing, we changed the survey window to ask about behaviors in the last seven days, as people had trouble recalling activities for 14 days. The survey was sent out in seven waves from May 19 to July 24. After an initial lock-down period, Ohio began to reopen on April 30; daily cases bottomed on June 15 and then rose substantially; mask-wearing was mandated on July 23 (Figure 1) [11].

Survey responses before and after the June 15 nadir were compared using student’s t-tests for numeric variables and the Pearson chi-square or Fisher’s exact tests for categorical variables. Secondarily, we compared respondents who stated they “always wore masks” to respondents who wore masks less often to identify if there were other differences in public health behaviors. All statistical tests were two-tailed with a significance threshold of 0.05. Analyses were conducted using R v.4.02. This work was approved by the Cleveland Clinic Institutional Review Board. An earlier version of this article was published as a preprint [12].

## Results

A total of 654 individuals responded (7% response rate) and were similar in age and sex before and after the June 15 nadir in daily cases. The majority of respondents were female (69%) and the median age was 56 years. A majority of respondents wore a mask outside the home, 53% before and 64% after the nadir (p = 0.008). More respondents attended a gathering of 10 or more people after the nadir (19% versus 11%, p=0.01) (Table 1). Those who attended a large gathering were less likely to wear masks (34% versus 66%, p<0.001).

**Table 1:** Survey responses before and after the nadir of cases* *Survey responses were grouped into before the nadir of COVID-19 cases in Ohio (June 15, 2020) and after. Besides the demographic and health status questions, the survey asked respondents to use a seven-day look-back period for their activities. Values are given as number (%) or median (interquartile range) for age and number of people living at home.

Survey Question	Pre-nadir n=214 N (%)	Post-nadir n=440 N (%)	p-value
Close contact with someone who has COVID*	1 (0.5)	5 (1)	0.67
Demographics and health status			
Age	54 (37, 66)	56 (36, 70)	0.53
Gender - Female	139 (66)	304 (70)	0.33
Number of people living at home	2 (2, 4)	2 (2, 3)	0.41
Someone at home had URI symptoms in the past week	7 (3)	20 (5)	0.54
Someone at home provides patient care	24 (11)	38 (9)	0.35
Been placed in isolation or quarantine	9 (4)	8 (2)	0.11
Lung disease (e.g. asthma, chronic obstructive pulmonary disease)	30 (14)	61 (14)	1
Immune suppression	20 (9)	32 (7)	0.44
High blood pressure	65 (30)	127 (29)	0.76
Social activities			
Gone to a friend, neighbor, or relative's residence	108 (51)	211 (48)	0.64
Attended a large gathering (10+ people)	23 (11)	84 (19)	0.01
Gone out to a bar, club, or other place where people gather	26 (12)	66 (15)	0.38
Activities of daily living			
Gone to the grocery store or pharmacy	167 (79)	342 (78)	0.88
Sought care from a hospital or health care facility	35 (16)	95 (22)	0.13
Remained in your residence at all times, except for essential activities or exercise	122 (57)	243 (55)	0.72
Shared items like towels or utensils with other people	55 (26)	84 (19)	0.06
Had close contact (within 6 feet) with people who live with you	181 (85)	359 (83)	0.50
Had close contact (within 6 feet) with people who do not live with you	125 (58)	230 (53)	0.20
Gone outside to walk, hike, or exercise	182 (86)	351 (81)	0.16
Shared a household with someone who has been interacting with 10+ people/day.	35 (16)	103 (23)	0.05
Travel and work			
Used shared public transit (e.g. train, bus, or subway)	1 (0.5)	7 (2)	0.28
Used a ride-share service (e.g. Uber or Lyft) or Taxi.	2 (1)	8 (2)	0.51
Traveled by airplane	0 (0)	2 (0.5)	1
Worked or studied outside the home	55 (26)	118 (27)	0.83
Been exposed to 10 or more people per day through my work.	39 (18)	85 (19)	0.82
Cleaning			
ALWAYS washed my hands or used hand sanitizer after possible exposures	130 (62)	293 (67)	0.21
ALWAYS washed my hands for at least 20 seconds	157 (74)	304 (69)	0.28
ALWAYS cleaned and disinfected shared surfaces daily	92 (43)	183 (42)	0.87
ALWAYS cleaned or disinfected mail or groceries.	53 (25)	74 (17)	0.02
Risk avoidance			
ALWAYS worn gloves outside of my home	15 (7)	20 (5)	0.21
ALWAYS avoided leaving my home at all	21 (10)	36 (8)	0.58
ALWAYS avoided others in public spaces	76 (36)	177 (41)	0.29
ALWAYS avoided touching my face while in public spaces.	92 (44)	222 (51)	0.08
ALWAYS wore a mask when leaving home	113 (53)	281 (64)	0.008
Which type of mask did you wear?			
Cloth	141 (66)	329 (75)	0.02
Surgical	82 (38)	135 (31)	0.06
N95	22 (10)	41 (9)	0.80
Other	10 (5)	15 (3)	0.57

Changes in social behavior differed by age group. Attendance at gatherings of 10 or more people increased among 18-29-year-olds and 30-49-year-olds (by 25%, p=0.02, and 17%, p=0.05, respectively), but not among older patients. Mask usage increased among 50-64-year-olds (40% to 58%, p=0.03). Respondents over 65 had the highest mask usage during both time periods (69% to 70%). Activities such as grocery shopping, seeking healthcare, and hand-washing did not change over time in any group (Table 2).

**Table 2:** Activities of daily living, social activities, and risk avoidance before and after the nadir of cases by age group* *Survey responses were grouped into before the nadir of COVID-19 cases in Ohio (June 15, 2020) and after. Besides the demographic and health status questions, the survey asked respondents to use a seven-day look-back period for their activities.

	18-29			30-49			50-64			65+		
	Pre-nadir n=29 N (%)	Post-nadir n=74 N (%)	p-value	Pre-nadir n=56 N (%)	Post-nadir n=96 N (%)	p-value	Pre-nadir n=58 N (%)	Post-nadir n=110 N (%)	p-value	Pre-nadir n=62 N (%)	Post-nadir n=149 N (%)	p-value
Activities of Daily Living												
Gone to the grocery store or pharmacy	25 (86)	58 (72)	0.53	47 (84)	79 (82)	0.97	50 (88)	94 (86)	0.98	39 (64)	104 (70)	0.51
Sought care from a hospital or health care facility	8 (28)	15 (20)	0.59	8 (14)	26 (27)	0.10	6 (10)	22 (20)	0.16	12 (19)	28 (19)	1
Remained in your residence at all times, except for essential activities or exercise	12 (41)	25 (34)	0.62	36 (64)	46 (48)	0.07	26 (45)	61 (55)	0.25	44 (71)	104 (70)	0.98
Had close contact (within 6 feet) with people who do not live with you	19 (66)	56 (76)	0.43	31 (55)	54 (57)	0.99	40 (69)	59 (54)	0.09	31 (50)	57 (39)	0.18
Social Activities												
Gone to a friend, neighbor, or relative's residence	17 (59)	53 (72)	0.30	30 (54)	48 (50)	0.80	35 (60)	46 (42)	0.03	22 (35)	59 (40)	0.64
Attended a large gathering (10+ people)	3 (10)	26 (35)	0.02	5 (9)	22 (23)	0.05	9 (16)	14 (13)	0.79	5 (8)	19 (13)	0.48
Gone out to a bar, club, or other place where people gather	5 (17)	11 (15)	0.77	5 (9)	20 (21)	0.09	12 (21)	24 (22)	1	2 (3)	9 (6)	0.51
Cleaning												
ALWAYS washed my hands for at least 20 seconds	19 (66)	50 (68)	1	42 (75)	68 (72)	0.79	40 (69)	70 (64)	0.60	50 (81)	106 (71)	0.21
ALWAYS cleaned or disinfected mail or groceries.	9 (31)	8 (11)	0.02	10 (18)	20 (21)	0.79	14 (25)	13 (12)	0.06	14 (24)	28 (19)	0.69
Risk Avoidance												
ALWAYS avoided leaving my home at all	0 (0)	2 (3)	1	3 (5)	5 (5)	1	4 (7)	5 (5)	0.49	11 (18)	23 (16)	0.87
ALWAYS wore a mask	16 (55)	47 (64)	0.58	26 (46)	58 (60)	0.13	23 (40)	64 (58)	0.03	43 (69)	104 (70)	1

Respondents who always wore masks were more often female (75% versus 59%, p <0.001), and they practiced other protective behaviors. Those who always masked washed hands more frequently after possible exposures (78% versus 46%, p<0.001) and avoided others in public spaces (51% versus 22%, p<0.001). Respondents who did not always mask outside their home were more likely to visit a friend or neighbor’s residence (58% versus 43%, p<0.001) or a bar or club (24% versus 8%, p<0.001) and have close contact with people that they did not live with (70% versus 45%, p<0.001).

## Discussion

In this repeated cross-sectional survey study, we found an increasing percentage of individuals attended large gatherings as cases were rising in Ohio, compared to a period of declining cases. Increased socialization was most apparent among 18-29-year-olds, possibly illustrating ‘caution fatigue’ amongst those at the lowest risk of dying from COVID-19 [13]. This behavior has been reported anecdotally in the lay press but has not been previously documented. As colleges begin to reopen around the country, there are growing concerns about potential outbreaks on campuses. Our study adds to these concerns, as we found that this age group is most likely to disregard public health recommendations and gather in large groups.

While the majority of respondents, especially those over 65 years, used masks, individuals who attended gatherings did so less consistently. As our survey was distributed prior to mask mandates in Ohio, we did not capture the impact of these mandates on public behavior. Essential activities and sanitization practices remained largely consistent across time and age groups, although the percentage of respondents who routinely disinfected groceries or mail decreased over time. This may reflect increased awareness of public health messaging, as the Centers for Disease Control and Prevention (CDC) states there is a very low risk of spread from these objects [14]. The majority of respondents practiced hand-washing after possible exposures, suggesting that this may have become a habit that will persist throughout the pandemic and afterward.

There have been concerns that those who wear face coverings may be less likely to adopt other protective measures due to risk compensation. Our study suggests the opposite, that those who wear masks are more likely to follow all public health recommendations. Conversely, we found that those who did not wear masks were less likely to adhere to social distancing recommendations, which may contribute to the further spread of the virus. Our study supports findings from a recent review, which concluded that face masks do not adversely affect hand hygiene [15].

The relationship between age and mask-wearing suggests that people generally wear masks to protect themselves, as mask-wearing was most common among those at the highest risk. This is understandable but may represent a failure of public health messaging. Cloth masks, which were worn by the majority of our respondents, are not completely effective at protecting the wearer, especially if they are in the vicinity of individuals not wearing masks. Therefore, protecting vulnerable patients solely by having them wear cloth masks will be less effective. Instead, masks should be worn by everyone to prevent spread by asymptomatic carriers. Since younger people are most likely to have asymptomatic infections, it is important that they wear masks. However, it may be difficult for people to think of themselves as a source of infection when they feel well. For example, a study of physicians found that hand washing was much more common when leaving a patient’s room than when entering [16]. Replacing such natural tendencies for self-preservation with altruistic behaviors will likely require social pressure. Mask mandates could be helpful in that regard.

Study limitations include sampling from a population that has accessed the healthcare system in the past year and may be more aware of public health messaging. There may also be a non-response bias, as the survey was computer-based.

## Conclusions

As the national conversation focuses on safe economic revival, it appears important to limit gatherings of more than 10 people and encourage mask-wearing. Essential activities and sanitization practices have not changed across time, and these practices are not affected by mask-wearing. Messaging should target younger patients, who are also least likely to wear masks. This may become increasingly important as colleges welcome students back to campuses, where social distancing may be harder to practice.
